# Magnetoionics for Synaptic Devices and Neuromorphic Computing: Recent Advances, Challenges, and Future Perspectives

**DOI:** 10.1002/smsc.202400133

**Published:** 2024-07-04

**Authors:** P. Monalisha, Maria Ameziane, Irena Spasojevic, Eva Pellicer, Rhodri Mansell, Enric Menéndez, Sebastiaan van Dijken, Jordi Sort

**Affiliations:** ^1^ Departament de Física Universitat Autònoma de Barcelona Cerdanyola del Vallès 08193 Bellaterra Spain; ^2^ NanoSpin Department of Applied Physics Aalto University School of Science FI‐00076 Aalto Finland; ^3^ Institució Catalana de Recerca i Estudis Avançats (ICREA) Pg. Lluís Companys 23 08010 Barcelona Spain

**Keywords:** artificial synapses, brain‐inspired memories, magnetoionics, skyrmions

## Abstract

With the advent of Big Data, traditional digital computing is struggling to cope with intricate tasks related to data classification or pattern recognition. To mitigate this limitation, software‐based neural networks are implemented, but they are run in conventional computers whose operation principle (with separate memory and data‐processing units) is highly inefficient compared to the human brain. Brain‐inspired in‐memory computing is achieved through a wide variety of methods, for example, artificial synapses, spiking neural networks, or reservoir computing. However, most of these methods use materials (e.g., memristor arrays, spintronics, phase change memories) operated with electric currents, resulting in significant Joule heating effect. Tuning magnetic properties by voltage‐driven ion motion (i.e., magnetoionics) has recently emerged as an alternative energy‐efficient approach to emulate functionalities of biological synapses: potentiation/depression, multilevel storage, or transitions from short‐term to long‐term plasticity. In this perspective, the use of magnetoionics in neuromorphic applications is critically reviewed, with emphasis on modulating synaptic weight through: 1) control of magnetization by voltage‐induced ion retrieval/insertion; and 2) control of magnetic stripe domains and skyrmions in gated magnetic thin films adjacent to solid‐state ionic supercapacitors. The potential prospects in this emerging research area together with a forward‐looking discussion on future opportunities are provided.

## Graphical abstract

The utilization of magnetoionics in neuromorphic applications is revised. Two ways to modulate synaptic weights are presented as “case studies”: 1) tuning of magnetization by voltage‐induced ion motion and 2) control of magnetic stripe domains and skyrmions in gated magnetic films grown adjacent to solid‐state ionic supercapacitors. The potential prospects and future research directions in this emerging research area are discussed.

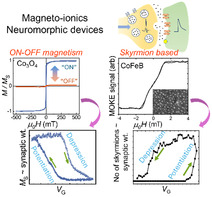

## 1 Introduction

### 1.1 Basic Concepts and Historical Background

Over the last decades, bioinspired materials have emerged to facilitate the transition toward greener and more sustainable electronics. Nature's models are often the source of sophisticated engineering designs. One of the most compelling ideas is to establish pathways to emulate the working principle of the biological brain. In particular, the human brain can learn, perceive, and store information in a highly energy‐efficient manner, outperforming conventional computers in tasks such as pattern recognition, data classification, language processing, adaptation, or prediction, all of them very important in the new era of Big Data.

In recent years, software‐based artificial neural networks derived from the original concept of the “perceptron” (i.e., a supervised learning algorithm)^[^ ^1^ ^]^ have been developed. However, neuromimetic software still uses digital computers with advanced central processing units and graphics cards that spend huge amounts of energy and resources.^[^ ^2^, ^3^ ^]^ Progress in neuromorphic hardware is slow because of: 1) the incomplete understanding of the complexity of biological brain; and 2) the limited capability of currently available materials and devices to mimic the massive interconnections and the basic functionalities of neurons and synapses. A milestone in the implementation of physical artificial neural networks was achieved in 2014 using CMOS processors, when IBM developed the TrueNorth hardware, that comprised 1 million artificial spiking neurons with 256 million configurable synapses. Yet, this neuromorphic processor lacked real‐time learning capability and it had important drawbacks in terms of cost and energy consumption associated with the extremely large number of transistors required for its operation.^[^ ^4^, ^5^ ^]^ A more sophisticated option includes, for example, reservoir computing, where the internal dynamics of a material system replaces discretely defined neurons and synapses in a predefined array. This machine learning framework is suitable for handling and forecasting time‐evolving data based on transforming time‐series inputs into spatiotemporal patterns, requiring training only on the readout signals.^[^ ^6^ ^]^

### 1.2 Brain‐Inspired Materials and Synaptic Devices

One of the reasons why conventional computers lack efficiency is that they comprise separate storage and data‐processing units, which need to be in continuous communication during computation, thus spending huge amounts of time and energy (von Neumann bottleneck). In contrast, the biological brain is able to perform “in‐memory computing”. The neurons and synapses can simultaneously retain and manipulate information through electrochemical signals called “action potentials”.

In the human brain, the synapses are functional connections between adjacent neurons, responsible for a large part of the learning and memory processes. The signal transmission across a presynaptic and postsynaptic neuron is mediated through ion migration. The strength of this connection is called the “synaptic weight”. Synapses have the capability to change their weight depending on the activity level, that is, strengthening under more action potentials and *vice versa*, leading to synaptic plasticity. Remarkably, there are combinations of materials that can emulate this kind of behavior to some extent. These include:^[^ ^7^, ^8^, ^9^, ^10^, ^11^, ^12^, ^13^, ^14^, ^15^, ^16^, ^17^, ^18^, ^19^, ^20^ ^]^ 1) two‐terminal devices such as resistive switching elements (where switching between low‐ and high‐resistance states stems from ionic migration along filamentary paths that traverse the metal–insulator–metal stacks), phase change materials, spin‐transfer torque (STT) nano‐oscillators, ferroelectric switching elements; and 2) three‐terminal devices such as ferroelectric field‐effect transistors, electrochemical random‐access systems, spin‐orbit torque magnetic switches, or memtransistors incorporating 2D semiconductors.

Memristors^[^ ^21^, ^22^, ^23^ ^]^ (based on, e.g., TaO_*x*_ or HfO_2_) are advantageous since they exhibit analog variations of electric conductivity with voltage, nonlinearity, high endurance, and operation in μs or faster (as required in brain‐inspired computing and, in particular, deep neural networks). Embedding memristor devices in crossbar arrays has been a widely employed method to create artificial neural networks.^[^ ^24^, ^25^, ^26^, ^27^, ^28^ ^]^ Nanoionic resistive switching materials exhibit rich internal ionic dynamics that enables them to operate with intrinsic data‐processing capabilities.^[^ ^29^, ^30^ ^]^ In turn, nanoionic transistors (operating through, e.g., Li^+^ intercalation in LiCoO_2_), in which electrochemical effects are utilized to control the channel resistance, can be used in reservoir computing.

### 1.3 Magnetic Materials for Neuromorphic Computing

An interesting class of materials for neuromorphic computing are magnetic materials.^[^ ^31^ ^]^ Stacks of nanoscale magnetic multilayers functioning as STT nano‐oscillators have been found suitable to emulate “spiking neural networks” that comprise integrate‐and‐fire neurons interconnected through stochastic, binary synapses.^[^ ^32^ ^]^ Spin Hall nano‐oscillators, where voltage is used to tune the frequency of the oscillations (through voltage‐controlled magnetic anisotropy), have been also proposed.^[^ ^33^, ^34^ ^]^

In general, the use of magnetism for brain‐inspired computing is appealing since magnetic materials are at the heart of data centers and write/read heads in conventional computers. Thus, there is potential for magnetic materials to become key players when attempting to merge current memory technologies with brain‐inspired computing. Spintronics‐based devices are very appealing, thanks to their nonvolatility, remarkable scalability, and fast operation speed.^[^ ^35^ ^]^ However, conventional memory device designs rely only on two binary states (digital output). This can be a limitation in neural networks requiring nonlinearity or synaptic plasticity. Thus, integration of memristors (with inherent synaptic weights) into spintronic devices appears to be a natural progression in the pursuit of advanced neuromorphic technologies.

Different pathways have been explored for the implementation of nonbinary magnetic synapses for deep learning. Memristive states achieved through domain wall motion have been reported in perpendicular‐anisotropy magnetic tunnel junctions.^[^ ^36^, ^37^ ^]^ In this kind of device, the change in the domain wall position alters the synaptic weight. Analog racetrack memories can also modulate the synaptic weight through the motion of domain walls.^[^ ^38^ ^]^ Skyrmions (i.e., topologically stable swirling‐spin quasiparticles that can be used as information careers with high endurance and speed) are also suitable for spintronic neuromorphic hardware.^[^ ^39^, ^40^ ^]^ Current‐driven generation, motion, and annihilation of skyrmions have been employed to mimic potentiation and depression behaviors.^[^ ^41^ ^]^ The size and density of skyrmions in certain areas can emulate synaptic weights, typically measured by tunneling magnetoresistance or the anomalous Hall effect.^[^ ^41^, ^42^ ^]^ Skyrmion‐based spiking neuron concepts taking advantage of interactions between skyrmions and applied currents, geometric constrictions, or other neighboring skyrmions have also been proposed.^[^ ^43^, ^44^ ^]^ The nonlinear dynamics of skyrmions has been further exploited in reservoir computing systems for pattern recognition, forecasting, adaptive processing, and other tasks.^[^ ^45^, ^46^, ^47^, ^48^, ^49^, ^50^ ^]^ Magnetic metamaterials comprising arrays of interacting magnetic nanorings (showing a mixture of vortex, onion, and states with 90° domain walls) have also found application in reservoir computing. In these materials, application of magnetic field triggers changes among the states, resulting in variations of anisotropic magnetoresistance that can be harnessed in different ways to create reservoirs with different computational capabilities.^[^ ^51^ ^]^

### 1.4 Voltage‐Controlled Magnetic Synapses and the Emergence of Magneto‐Ionic Synapses

The utilization of magnetic fields (generated through miniaturized electromagnets) or electric currents is not energy efficient since a large fraction of incoming power is dissipated through Joule heating effects.^[^ ^52^, ^53^, ^54^ ^]^ Therefore, implementation of magnetic synapses directly controlled with voltage (with minimized flowing currents) is highly desirable. Voltage can be used to drive domain wall motion in racetrack‐like memories^[^ ^55^, ^56^, ^57^ ^]^ or in magnetic tunnel junctions with voltage‐controlled perpendicular magnetic anisotropy (PMA).^[^ ^58^, ^59^, ^60^ ^]^ In turn, skyrmions can be manipulated with voltage through different mechanisms: accumulation of interfacial electrostatic charges in ultrathin magnetic films,^[^ ^61^, ^62^, ^63^, ^64^, ^65^, ^66^, ^67^ ^]^ strain transfer from piezoelectrics,^[^ ^68^, ^69^ ^]^ and locally applied electric fields.^[^ ^70^ ^]^ These approaches offer prospects for low‐power reservoir computing (with ≈fJ per input event). Remarkably, voltage control of Ruderman–Kittel–Kasuya–Yosida (RKKY) interactions in CoFeB synthetic antiferromagnets could be also used to modulate the size of skyrmions and, concomitantly, the resistance (synaptic weight).^[^ ^71^ ^]^

In recent years, modification of the magnetic properties of materials has been also accomplished *via* voltage‐driven ion motion. This approach, referred to as “magneto‐ionics,” merges memristor effects with magnetism, has brought unprecedented results in inducing nonvolatile changes of magnetic anisotropy,^[^ ^72^, ^73^, ^74^, ^75^ ^]^ full ON–OFF (ferromagnetic–paramagnetic) transitions in metal–oxide and nitride films,^[^ ^76^, ^77^, ^78^ ^]^ tuning of exchange bias,^[^ ^79^, ^80^, ^81^ ^]^ or control of domain walls and skyrmionic states,^[^ ^82^, ^83^, ^84^ ^]^ among others. Different ion species (O^2−^, N^3−^, H^+^, Li^+^, etc.) have been used to trigger these effects.^[^ ^74^ ^]^ Given its resemblance to memristors, magnetoionics has been proposed as a means to build arrays of artificial synapses.^[^ ^85^ ^]^ Similar to biological synapses, magnetic weights can be induced in these devices through voltage pulses in an energy‐efficient manner.

Several recent studies have shown that magnetoionic devices are suitable to mimic potentiation and depression functions, spike‐amplitude and spike‐timing dependences, nonlinearity, threshold voltages, learning and forgetting capabilities, or transition from short‐term (STP) to long‐term plasticity, among other synaptic effects.^[^ ^85^, ^86^, ^87^, ^88^, ^89^ ^]^ Particularly interesting is the emulation of “learning under deep sleep” caused by interface N^3−^ ion accumulation in thin transition‐metal films previously actuated with voltage at high frequencies.^[^ ^87^ ^]^ In this perspective, we demonstrate some synaptic functionalities using Co_3_O_4_ as synaptic thin films (case study I). In a separate section (case study II), we overview the potential of magnetoionics to tune the properties of magnetic domain walls and skyrmions,^[^ ^84^ ^]^ such as their position, size, and density, all of which can be used to modulate the synaptic weight. We then discuss the potential of magnetoionic devices for neuromorphic computing both as synapses and more generally.

## 2 Case Study I: Magnetoionic Single‐Layer Synapses

### 2.1 Thin‐Film Growth, Magneto‐Ionic Actuation, and Working Principle

Co_3_O_4_ thin films of different thicknesses (7 and 15 nm) were grown on [100]‐oriented Si substrates previously metalized with Ti (20 nm)/Cu (60 nm) seed layers. The films were deposited using an AJA International ATC 2400 sputtering system at room temperature. The base pressure was ≈10^−8^ Torr. The oxide films were reactively grown in an oxygen environment (Ar:O_2_ = 10:1) with a pressure of 3 × 10^−3^ Torr and at a growth rate of 3.3 Å s^−1^. A part of the substrate was masked with Kapton tape during deposition, and the exposed Ti/Cu bilayer was used to make the electrical connection for the magnetoionic actuation.

**Figure 1:**
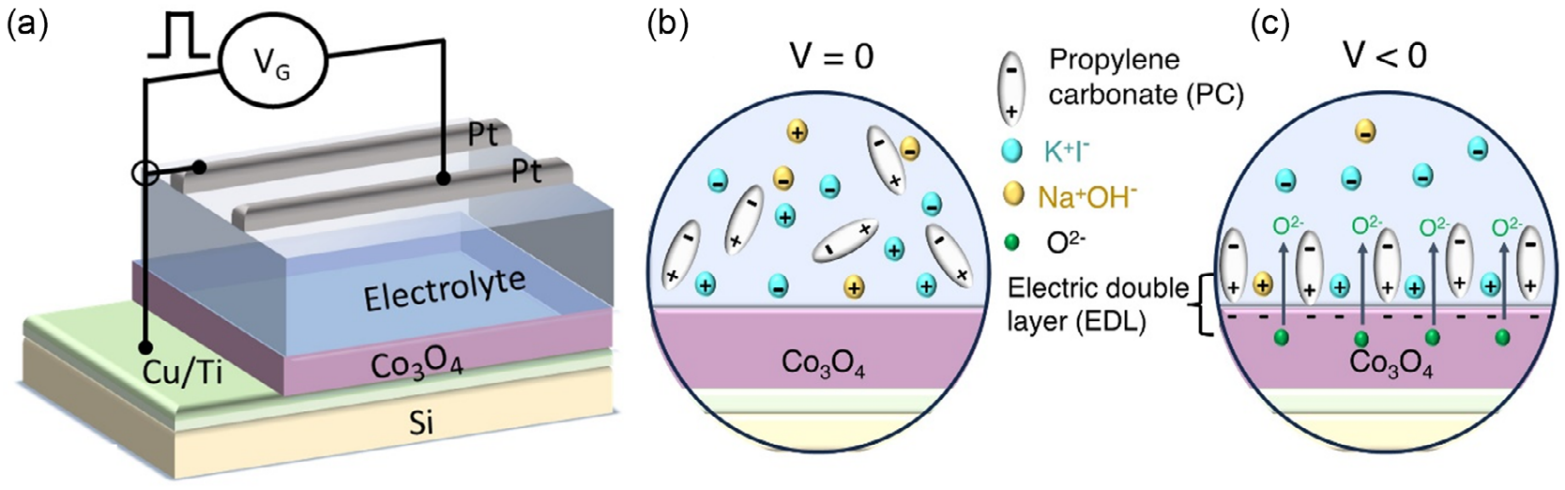
a) Schematic illustration and electrical connection of the Co_3_O_4_‐based magnetic synapse in capacitor configuration. b) Schematics of the ionic distribution in the electrolyte at no‐gating condition. c) Schematics to illustrate the physical mechanism governing magnetoionics with extraction of oxygen ions upon negative gating.

All the magnetoelectric measurements were carried out using a vibrating sample magnetometer (VSM) from Micro Sense LOT‐Quantum design at room temperature. The magnetoionic synapse was mimicked by a three‐electrode device (**Figure** **1**a) in a capacitor‐like configuration, where the bottom Ti/Cu bilayer was used as the working electrode and two Pt wires served as the counter and the reference electrode. An anhydrous propylene carbonate (PC) was used as the gate electrolyte. Metallic Na pieces were introduced into the PC solvent to eliminate traces of residual water in the electrolyte, thereby generating Na^+^ and OH^−^ ions. The concentration of resulting Na^+^ ions was in the range 10–25 ppm. To increase the ionic strength of the solvent, 2.5 × 10^−4^ M potassium iodide (KI) was added to the electrolyte.^[^ ^90^ ^]^ All the in situ magnetic measurements were carried out in the VSM using an in‐plane geometry. A high‐field straight‐line correction was made to subtract the diamagnetic/paramagnetic contributions from the substrate and holder. The gate voltage pulses were applied using Agilent B2902A power supply, using Labview programming.

The working mechanism of the magnetoionic device is presented in Figure 1b,c. At no‐gating condition, the ions and molecules in the electrolyte are distributed randomly (Figure 1b). With voltage application (in this case negative voltage), an electric double layer (EDL) is formed at the interface between the sample and electrolyte, creating a strong electric field (Figure 1c). The K^+^ and Na^+^ ions dissolved in the electrolyte approach the sample by Coulombic attraction, thereby increasing the ionic strength of the EDL. It is expected that, upon negative gating, the strong electric field drives O^2−^ ions out of the crystal lattice, causing Co_3_O_4_ reduction and formation of metallic Co.^[^ ^76^, ^86^ ^]^ Conversely, with positive gating, the O^2−^ ions are inserted back into the Co_3_O_4_ lattice assisted by the positive electric field, causing reoxidation of previously formed Co clusters. Hence, the working mechanism involves both electrostatic and electrochemical effects.^[^ ^53^, ^91^, ^92^ ^]^ Films of different thicknesses have been studied to take advantage of both volatile and nonvolatile magnetoionic effects to mimic synaptic functionalities broadly.

**Figure 2:**
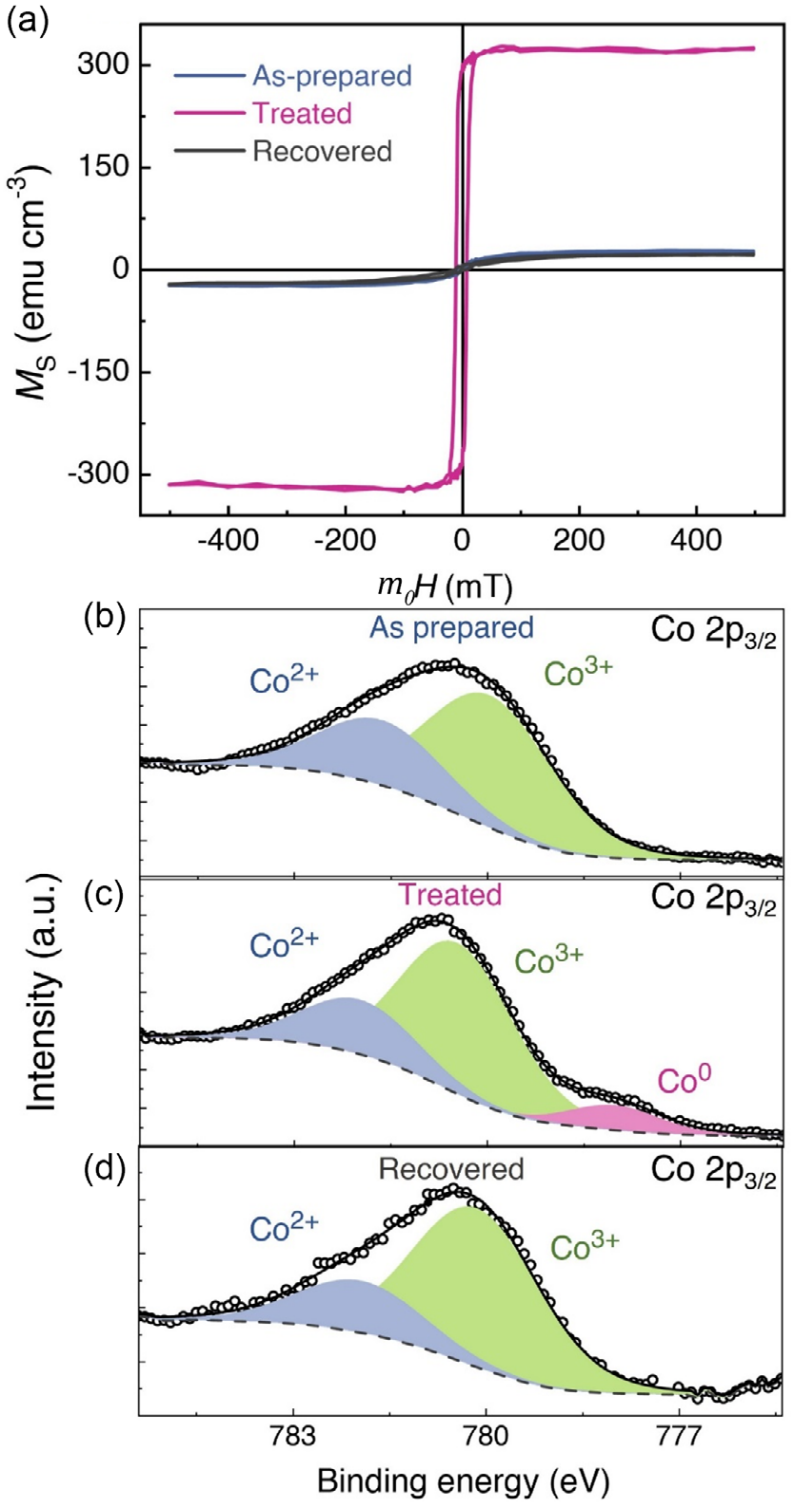
a) *M*–μ_0_ *H* hysteresis loops of 15 nm Co_3_O_4_ film in as‐prepared, treated (with *V*_G_ = –5 V for 1 h), and recovered (treated with *V*_G_ = 5 V for 1 h) states. The overlapping of *M*–μ_0_ *H* loops of as‐prepared and recovered sample shows complete reversibility. X‐ray photoelectron spectroscopy analysis of 15 nm Co_3_O_4_ film is shown in panels b–d): b) Co 2*p*_3/2_ spectra of the as‐prepared sample consisting of Co^2+^ and Co^3+^ peaks. c) Co 2*p*_3/2_ spectra of the treated (*V*_G_ = –5 V, for 1 h) sample consisting of Co^0^ (metallic cobalt), Co^2+^, and Co^3+^ peaks. d) Co 2*p*_3/2_ spectra of the recovered (*V*_G_ = 5 V, for 1 h), for which the Co^0^ metallic contribution vanishes, and only Co^2+^ and Co^3+^ peaks are again identified.

The *M*–μ_0_ *H* loops of the as‐prepared, treated, and recovered samples gated at *V*_G_ = –5 V for 1 h and subsequently recovered at *V*_G_ = 5 V for 1 h are shown in **Figure** **2**a. While the as‐grown sample is virtually paramagnetic (with a tiny magnetic moment probably arising from the contribution of residual Co clusters formed during reactive sputtering), the film gated at negative voltage shows a clear hysteresis loop with saturation magnetization, *M*_S_, approaching 300 emu cm^−3^. Note that the applied voltage to induce the onset of ferromagnetic behavior (i.e., *V*_G_ = –5 V) is above the threshold voltage for which we observe magnetoionic effects in this kind of system when KI is added to PC in suitable concentrations and below the solubility limit (*V*_G,threshold_ ≈ –1.5 V);^[^ ^90^ ^]^ otherwise, *V*_G,threshold_ for Co_3_O_4_ films is around –8 V.^[^ ^77^ ^]^

The physical working mechanism responsible for the magnetic switching in the cobalt oxide thin films was probed using X‐Ray photoelectron spectroscopy (XPS). XPS characterization was carried out using Phoibos 150 analyzer, using a monochromatic Al Kα (*E* = 1486.6 eV) source. The XPS measurements were carried out ex situ immediately after voltage treatment of the samples. The data was charge referenced to C 1*s*, C–C peak at 284.8 eV. The XPS data was analyzed using CasaXPS software and a Shirley background was used for all the peak fittings. XPS measurements were performed on the 15 nm Co_3_O_4_ thin film due to its highly nonvolatile magnetic switching (that will be described in the following section). Figure 2b–d shows the core‐level Co 2*p*_3/2_ spectra of the thin film under different conditions. The XPS pattern of the as‐prepared thin film consists of Co^2+^ and Co^3+^ peaks as shown in Figure 2b. Meanwhile, the XPS pattern of the treated film (with *V*_G_ = –5 V for 1 h) consists of an additional Co^0^ peak along with Co^2+^ and Co^3+^ peaks, as shown in Figure 2c, confirming the partial electrochemical reduction (extraction of O^2−^ ions) of the film. After subsequent positive voltage treatment with *V*_G_ = 5 V (for 1 h), the film shows again only Co^3+^ and Co^2+^ peaks, without any hint of Co^0^, as evidenced in Figure 2d, thereby corroborating reinsertion of O^2−^ ions into the material. Thus, the as‐prepared and recovered films show similar chemical composition, indicating good reversibility of the magnetoionic effects.

### 2.2 The Role of Film Thickness on Synaptic Potentiation: Volatile versus Nonvolatile Magnetoionic Effects

**Figure 3:**
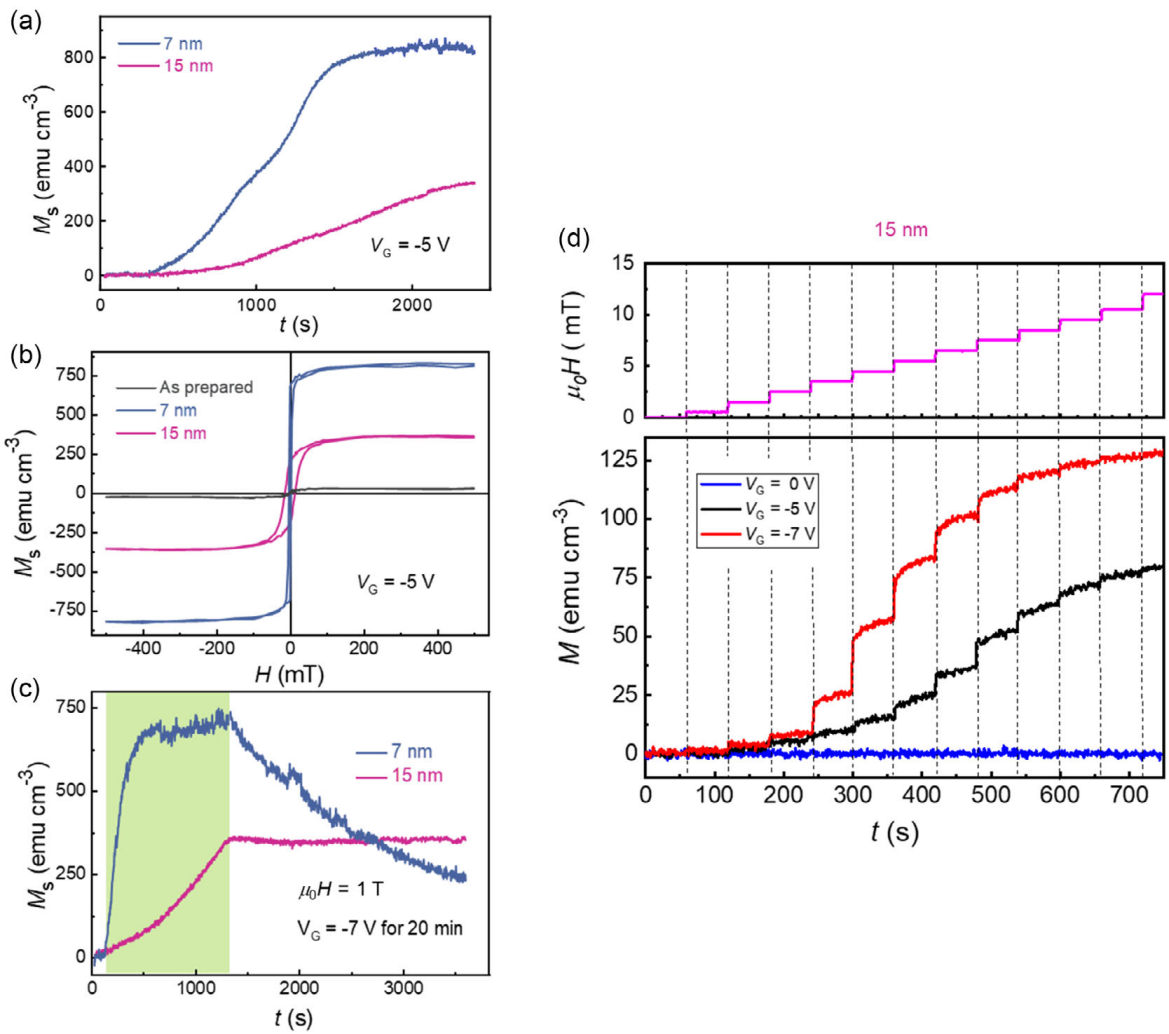
a) *M* versus *t* curves of 7 and 15 nm Co_3_O_4_ thin films treated with –5 V and measured by applying in‐plane magnetic field of μ_0_ *H* = 1 T. b) *M*–μ_0_ *H* hysteresis loops of both 7 and 15 nm films previously treated with –5 V for 40 min, showing a paramagnetic‐to‐ferromagnetic (OFF/ON) switching. Note that voltage was continuously applied during the measurement of the hysteresis loops. c) *M*–*t* curves of 7 and 15 nm Co_3_O_4_ thin films treated with *V*_G_ = –7 V for 20 min (green shadowed area), showing that the 7 nm film shows a volatile change of *M* after the voltage is turned off, whereas the 15 nm film exhibits a nonvolatile magnetoionic change. d) Dependence of *M* as a function of time for the 15 nm‐thick Co_3_O_4_ thin film measured at 0 V under different μ_0_ *H* values (top panel), after having subjected the samples to prior voltage treatments using *V*_G_ = 0, −5, and −7 V for 20 min and subsequent demagnetization processes with an alternating magnetic field of decreasing amplitude (AC degauss).

The time evolution of the magnetization (*M*) during the voltage treatment (*V*_G_ = –5 V) is shown in **Figure** **3**a for both 7 and 15 nm cobalt oxide films. An in‐plane magnetic field of 1 T was applied, which is much larger than the saturation field. *M* was found to drastically increase at the beginning of the treatments, tending to level off after some minutes. The magnetoionic speeds, calculated by linear fitting of the *M*–*t* curves in the first 1000 s, yield values around 0.80 and 0.15 emu cm^−3^ s^−1^ for 7 and 15 nm‐thick films, respectively. Remarkably, the magnetoionic rate is higher for the 7 nm film compared to the 15 nm film, in agreement with previous works.^[^ ^86^, ^87^ ^]^

The *M* versus μ_0_ *H* loops of the as‐prepared and voltage‐treated samples, measured at room temperature, are presented in Figure 3b. The loops are measured after voltage has been switched off; otherwise, hysteresis loops would show peculiar shapes (with, e.g., crossing ascending and descending branches), which would make the simultaneous role of voltage and magnetic field on the achieved magnetization difficult to be disentangled. With voltage treatment of –5 V, *M* increases drastically in both cases, showing a paramagnetic‐to‐ferromagnetic phase transition. *M*_S_ values of 754 and 295 emu cm^−3^ were attained for the 7 and 15 nm films after gating for 40 min. The coercivity (μ_0_ *H*_C_) was estimated to be 3 and 13.9 mT for the 7‐ and 15 nm‐thick film, respectively. A higher magnetoionic effect is observed in the thinner film owing to a stronger EDL effect at the film–electrolyte interface, leading to larger electric field across the CoO_*x*_ semiconducting films. These results corroborate that for negative voltage treatment, O^2−^ ions are extracted from the crystal lattice of the cobalt oxide, inducing a paramagnetic‐to‐ferromagnetic transition that gives rise to synaptic potentiation effects. The volatile and nonvolatile characteristics of the magneto‐ionic effects for the 7 and 15 nm films, respectively, are shown in Figure 3c. It can be seen that the thinner film has a tendency to be naturally re‐oxidize from the oxygen dissolved in the electrolyte, even in the absence of any positive voltage applied externally, thereby emulating short‐term synaptic plasticity. In the biological brain, the synapses are functional connections between adjacent neurons, and the signal transmission across presynaptic and postsynaptic neurons is mediated through the synapse *via* ion migration. Hence, these results confirm that magnetoionics (change in magnetic properties *via* ion motion) is a potential means to emulate a magnetic synapse for neuromorphic computing applications, where the sample *M* can be considered as the synaptic weight. Finally, Figure 3d shows the possibility of modulating the synaptic weight with magnetic field pulses. Several observations can be made from this experiment. First, if the sample is not actuated with voltage (*V*_G_ = 0 V), no change of *M* is observed even if magnetic field of growing intensity is applied (blue curve), as expected from the paramagnetic nature of the as‐grown films (note that a correction for the linear paramagnetic background is applied). To controllably tune *M* with μ_0_ *H*, the films were actuated with *V*_G_ = −5 V for 20 min, subsequently demagnetized with an AC magnetic field, and then *M* was measured as a function of time while applying magnetic field steps of growing intensity. A maximum *M* around 80 emu cm^−3^ was obtained after 750 s. If the process is repeated with *V*_G_ = −7 V, the changes in *M* at each magnetic field value become more pronounced. This indicates that larger negative *V*_G_ values generate larger fractions of ferromagnetic phase (by removal of O^2−^ ions). Thus, while *V*_G_ is responsible for the induced paramagnetic‐to‐ferromagnetic transition and the concomitant synaptic plasticity, the strength of the induced magnetic signal can be modulated also with the applied magnetic field.

**Figure 4:**
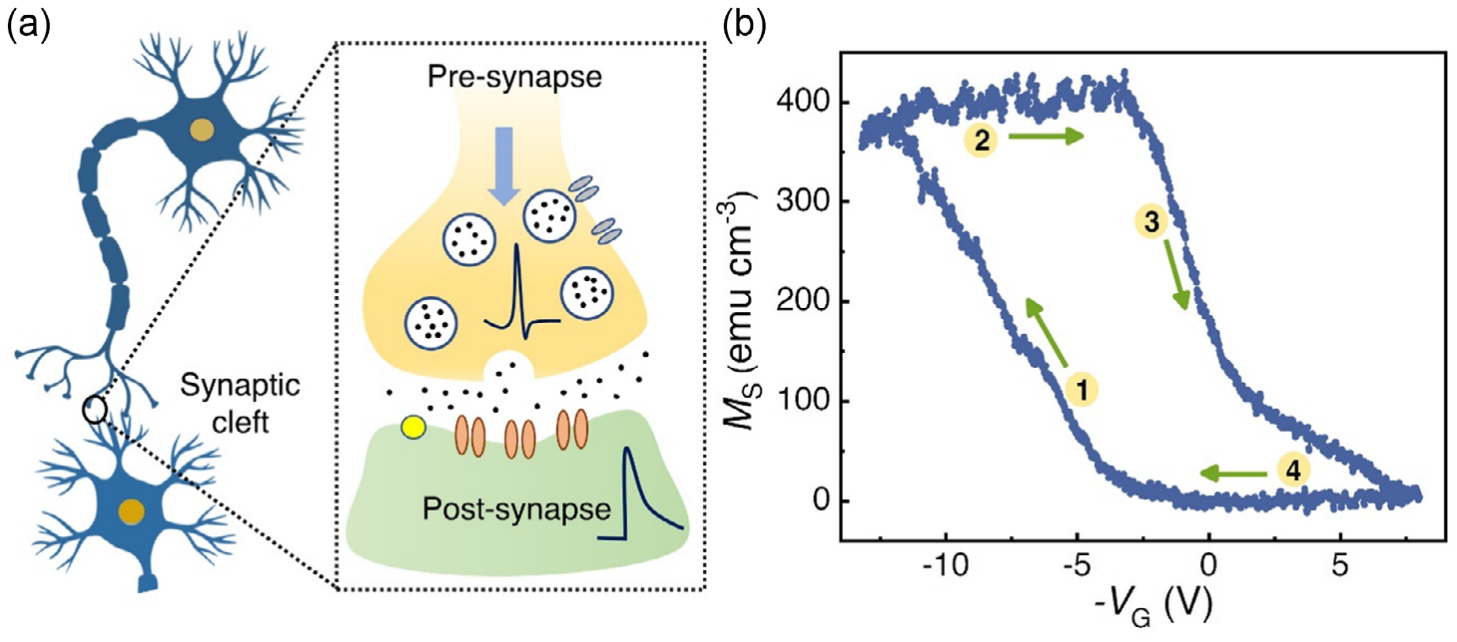
a) Schematics of signal transmission across a biological synapse by ion migration. b) The *M* versus. *V*_G_ curve of the 15 nm‐thick magnetic synapse shows nonvolatile and reversible change in sample *M* with *V*_G_ sweeping.

**Figure** **4**a shows the schematics of ion migration resulting in signal transmission in a biological synapse. Although magnetoionic effects for the 15 nm‐thick cobalt oxide film are nonvolatile, we show in Figure 4b that *M*_S_ can be both increased and subsequently decreased by application of DC voltage of first negative and then positive polarities. This graph emulates potentiation and depression effects under DC gating. Specifically, *V*_G_ was swept from 0 to –12 V, then from –12 to 7 V, and back to 0 V with a speed of 0.017 V s^−1^ in an anticlockwise chirality. During sweeping from 0 to –12 V (step ➊) *M* first increases to ≈430 emu cm^−3^ by extraction of O^2−^ ions from the Co_3_O_4_ crystal lattice. Then by sweeping from –12 to 0 V (step ➋), *M* reaches saturation and remains constant because further amount of O^2−^ cannot be pulled out from the material. However, with positive voltage treatment (sweeping from 0 to 7 V, step ➌), *M* reduces back to the initial virtually nonmagnetic state (inhibition behavior) by O^2−^ ion reinsertion into the crystal lattice. Finally, while sweeping from 7 to 0 V (step ➍), *M* remains constant and results in a closed *M* versus *V*_G_ loop. This giant loop opening in the *M* versus *V*_G_ curve represents nonvolatile and reversible magnetic switching, which is necessary to mimic synaptic functionalities in an electronic device.

### 2.3 Short‐Term Memory Effects in Thin Co_3_O_4_ Films

Synapses have the capability to change their strength (synaptic weight) depending on the activity level. This is defined as synaptic plasticity. Synaptic plasticity has been classified into two types: 1) STP; and 2) long‐term plasticity, depending on the retention time of the induced changes. STP is important for dynamical information processing, temporal encoding, and resource utilization. Long‐term plasticity is responsible for permanent learning and memory in the human brain, as well as for complex and long‐term cognitive tasks.^[^ ^93^ ^]^ As evidenced in section 2.2, and further described below, Co_3_O_4_‐based magnetic synapses can emulate both short‐term and long‐term plasticity by simply varying the thickness of the gated films, taking advantage of the thickness‐dependent volatile versus nonvolatile magnetoionic effect.

**Figure 5:**
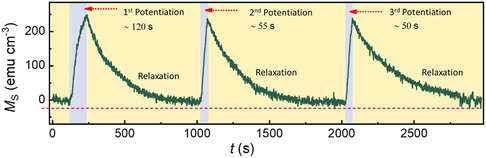
STP realized in the 7 nm Co_3_O_4_ thin film. Excitatory postsynaptic behavior has been emulated by applying negative gate voltage pulses (*V*_G_ = –7 V) allowing the sample to magnetically relax in between them (at *V*_G_ = 0 V). It is experimentally demonstrated that potentiation becomes easier upon the repeated application of a negative voltage in the thin Co_3_O_4_‐based artificial synapse.

To further assess the volatile character of magnetoionic effects in the 7 nm‐thick film, cyclic excitatory/relaxation behavior has been emulated in these magnetic synapses by applying negative gate voltage pulses (*V*_G_ = –7 V), as shown in **Figure** **5**. During pulse application, *M* increases to ≈250 emu cm^−3^, and upon pulse removal, the *M* relaxes back to the initial value, suggesting that O^2−^ ions travel back to the material to recover their initial state spontaneously, without any need of external stimuli.

The first potentiation is realized by applying the negative gate voltage pulse for 120 s. Afterward, *M* relaxes spontaneously to the initial state, akin to a synaptic depression process. Remarkably, a second potentiation to a similar Δ*M* value is achieved using the same gate voltage but with a shorter pulse duration (*t*_P_ = 55 s), demonstrating faster potentiation after the application of consecutive voltage pulses. The third potentiation process is even faster, as it is achieved within 50 s. These results suggest that the process of O^2−^ ion extraction from the crystal lattice becomes easier with repetitive action, indicating that atomic bonds may become looser and the films slightly less dense after voltage application, as observed in analogous magnetoionic systems.^[^ ^76^ ^]^

### 2.4 Long‐Term Memory Effects in Thick Co_3_O_4_ Films

**Figure 6:**
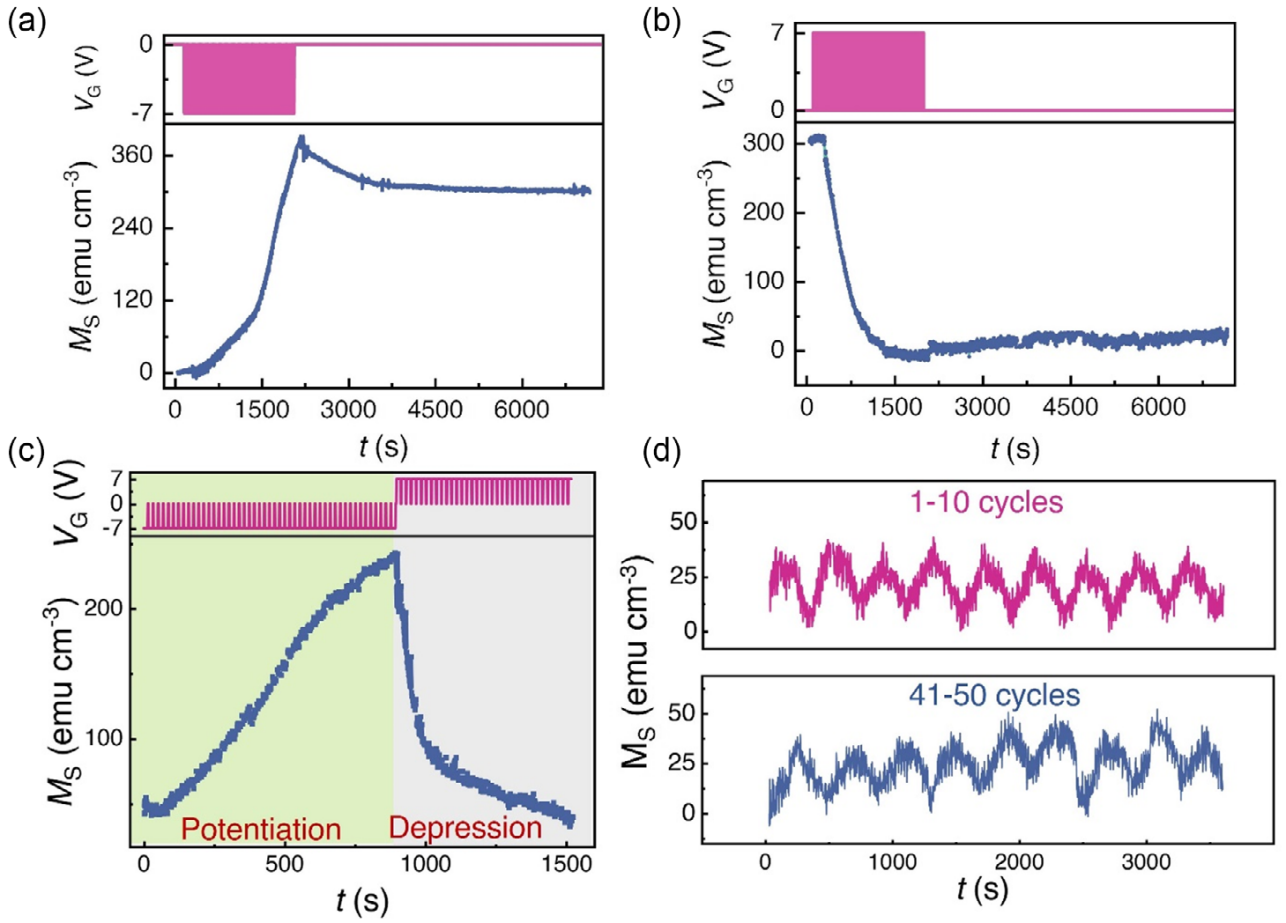
Long‐term plasticity realized in the 15 nm Co_3_O_4_ thin film with high retention. a) LTP: *M* progressively increases by applying a series of negative voltage pulses (*V*_G_ = –7 V, *t*_P_ = 15 s, *t*_D_ = 3 s); b) LTD: *M* decreases drastically by applying a series of positive voltage pulses (*V*_G_ = 7 V, *t*_P_ = 15 s, *t*_D_ = 3 s); c) simultaneous realization of one LTP + LTD cycle. d) Series of 50 potentiation/ depression cycles (see text for details) demonstrating good endurance of the effects.

Long‐term plasticity has been emulated using the 15 nm Co_3_O_4_ thin films. Long‐term potentiation (LTP), meaning a significant increment in synaptic weight (*M*), has been realized by applying a series of 100 negative gate voltage pulses (*V*_G_ = –7 V, *t*_P_ = 15 s, *t*_D_ = 3 s, where *t*_P_ and *t*_D_ denote the times while voltage was ON and OFF, respectively), as shown in **Figure** **6**a. The successive negative voltage treatment results in a drastic increment in *M*, that reaches ≈400 emu cm^−3^. Upon gate pulse removal, *M* decreases slightly but it retains a high value (it levels off and stays constant at around 300 emu cm^−3^ for the subsequent hour of measurement), evidencing that the larger thickness restricts a complete spontaneous re‐oxidation of the gated films. Similarly, long‐term depression (LTD), signifying a forced decrement in synaptic weight, has been experimentally demonstrated by applying a series of 100 positive gate voltage pulses (*V*_G_ = 7 V, *t*_P_ = 15 s, *t*_D_ = 3 s), see Figure 6b. After removal of pulses, the sample retains the same state (with virtually zero magnetization) for ≈1 h displaying high stability of the depressed state. The LTP and LTD can be realized simultaneously, as shown in Figure 6c. In this case, *M* increases by applying a series of negative gate voltage pulses (*V*_G_ = –7 V, *t*_P_ = 15 s, *t*_D_ = 3 s) and then is brought back to the initial state by applying a series of positive gate voltage pulses (*V*_G_ = 7 V, *t*_P_ = 15 s, *t*_D_ = 3 s). Note that the linearity of magnetization during the potentiation process is around 0.9. The cyclability of the device has been also checked by applying 10 consecutive negative voltage pulses (*V*_G_ = –7 V, *t*_P_ = 10 s, *t*_D_ = 10 s) followed by 10 consecutive positive voltage pulses (*V*_G_ = 7 V, *t*_P_ = 10 s, *t*_D_ = 10 s). To illustrate the endurance of the magnetic synapse, we show 50 potentiation/depression cycles under these conditions in Figure 6d.

**Figure 7:**
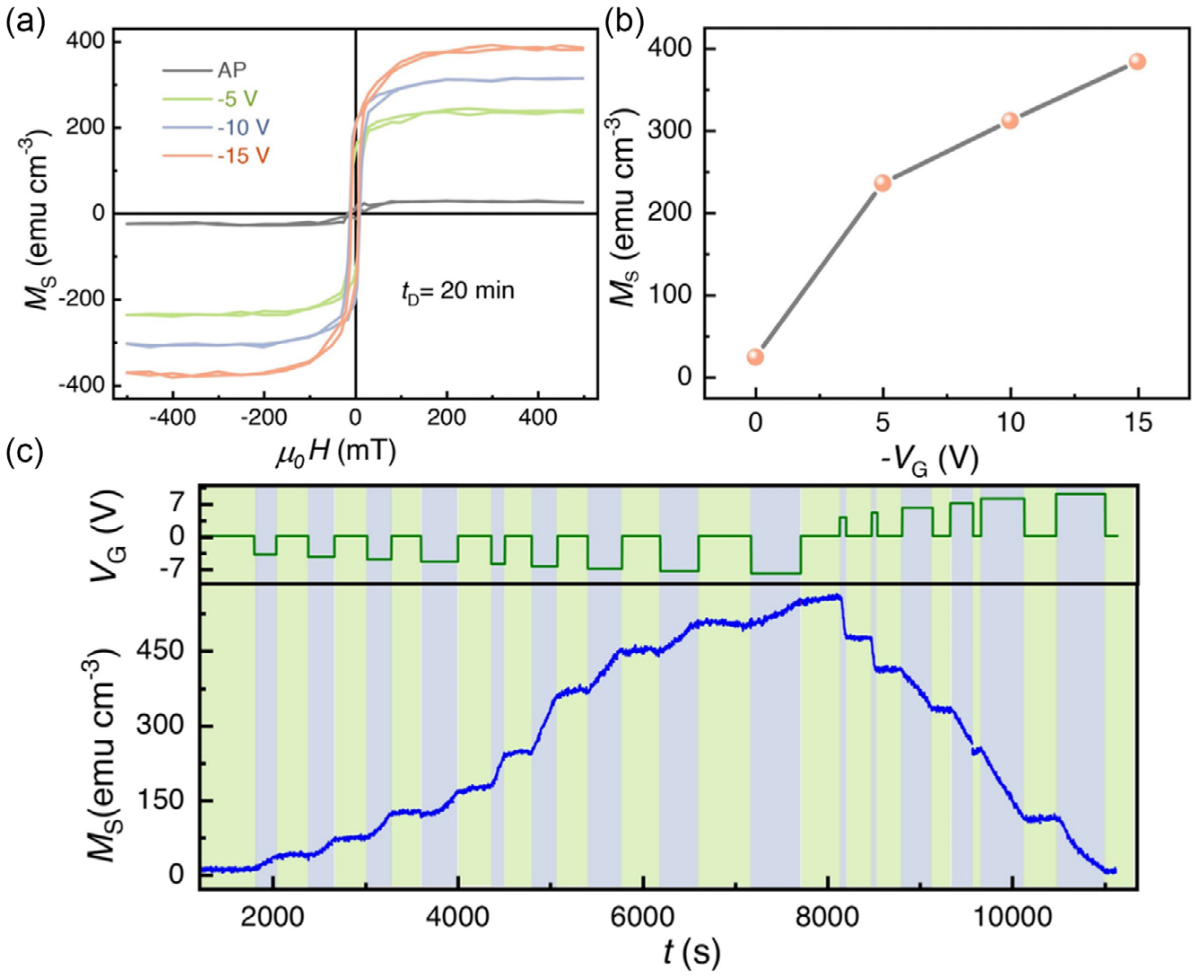
a) SADP: The *M*–μ_0_ *H* loops of 15 nm Co_3_O_4_ thin films treated with voltage pulses of different amplitudes (*V*_G_ = *–*5, *–*10, *–*15 V) for 20 min (“AP” corresponds to the as‐prepared state). b) Corresponding *M*_S_ values as a function of spike amplitude; c) Multilevel nonvolatile reversible magnetic states were realized for 15 nm Co_3_O_4_ thin film. A series of negative gate voltage pulses were applied to obtain a step‐wise increment of *M*. The magnetization was then brought back to the initial stage in a step‐wise manner by applying a series of positive gate voltage pulses.

The strength of synaptic weight can be tuned by varying the gate voltage amplitude, giving rise to spike‐amplitude‐dependent plasticity (SADP). SADP has been emulated in the 15 nm Co_3_O_4_ films by applying a series of slow voltage pulses of different amplitudes (0, *–*5, *–*10, *–*15 V) for a constant time (*t* = 20 min). The *M–*μ_0_ *H* loops of the treated samples are plotted in **Figure** **7**a, and they show a systematic increment in *M*_S_ with increasing spike amplitude, which is plotted in Figure 7b. Multilevel, nonvolatile, and reversible discrete magnetic states can be realized in the 15 nm Co_3_O_4_ thin films by applying different values of negative or positive gate voltage, due to the nonvolatile character of the magneto‐ionic effects. Multilevel states are crucial for analog computing and increased memory density. As shown in Figure 7c, multilevel states can be realized by applying a series of negative gate voltage pulses with increasing amplitude (from *–*4 to *–*8.5 V in steps of 0.5 V). During negative voltage application, the magnetization increases (shadowed in gray), and it remains constant during the zero‐voltage segments (shadowed in green), indicating its intrinsic nonvolatile nature. Furthermore, the magnetization can be brought back to the initial state by applying a series of positive voltage pulses (in this case, from 4 to 10 V in steps of 1 V). During the erasing process, we could obtain five distinct, nonvolatile states. The extraction/insertion of O^2−^ ions during the writing/erasing process with negative/positive voltage pulses resulted in 15 distinct multilevel states in total, nonvolatile, and suitable for neuromorphic applications. Note that analogous SADP effects have been also observed in CoFe_2_O_4_ 90 nm‐thick epitaxial nanopillars, although in that previous work a transition from STP to LTP was observed depending on the gating voltage.^[^ ^88^ ^]^

Finally, the resistance (*R*) of the as‐prepared and treated samples was measured using a four‐probe configuration on Co_3_O_4_ grown onto thermally oxidized Si substrates (in a transistor‐like configuration, without the Ti/Cu conductive underlayer).^[^ ^94^ ^]^ *R* was found to decrease by 26% from the as‐grown state when applying *V*_G_ = *–*15 V for 1 h suggesting that, with the extraction of more O^2−^ ions, the semiconducting Co_3_O_4_ layer becomes more metallic like. In parallel, the induced change of magnetization was also assessed in the transistor‐like configuration, leading to *M*_S_ = 45 emu cm^*−*3^ (as expected, much lower magnetization than in Co_3_O_4_ gated using the capacitor configuration^[^ ^94^ ^]^). These results provide evidence of a good correlation between changes in magnetic and electrical properties, illustrating that the memory state of the system might be read both magnetically and electrically. This can be exploited to design multifunctional synapses with both electrical and magnetic (i.e., spin) degrees of freedom.

## 3 Case Study II: Magnetoionic Synapses Based on Magnetic Domains and Skyrmions

### 3.1 Magnetoionic Supercapacitors with Voltage‐Controllable Spin Textures

Contrary to Case study I, which emulates the synaptic weight through magnetoionic control of the film magnetization, *M*, we now consider the use of reversible ion migration to tailor the magnetic microstructure of a thin film through changes of magnetic anisotropy and the Dzyaloshinskii–Moriya interaction (DMI). We focus on magnetic stripe domains and magnetic skyrmions in a thin CoFeB film with PMA. The synaptic weight, which is altered by small voltages, is represented by the number of stripe domains or skyrmions. Mimicking synaptic behavior using skyrmion states has been demonstrated in systems that are controlled by a magnetic field^[^ ^41^ ^]^ or film strain.^[^ ^95^ ^]^ Here, we consider a solid‐state Li‐ion supercapacitor structure to reversibly alter the magnetic structure of the CoFeB film. In previous works, we already showed that known materials from Li‐ion battery and supercapacitor technology such as LiCoO_2_ (LCO, a Li‐ion storage layer)^[^ ^96^ ^]^ and lithium phosphorous oxynitride (LiPON, a solid‐state electrolyte)^[^ ^97^, ^98^ ^]^ can be used to reversibly alter the direction of magnetization,^[^ ^75^ ^]^ the skyrmion density,^[^ ^84^ ^]^ or the strength and sign of RKKY interlayer coupling.^[^ ^99^ ^]^ A LCO/LiPON bilayer sandwiched between a magnetic electrode at the LiPON side and a gate electrode at the LCO side operates as solid‐state magnetoionic battery structure. In such multilayer stacks, large amounts of Li ions migrate between the LCO storage layer and the magnetic film at small voltage (≈2 V), inducing magnetic effects with record‐breaking magnetoelectric coupling efficiency.^[^ ^75^ ^]^ The timescale of voltage control in a magnetoionic battery is of the order 0.1–10 s. Alternatively, solid‐state Li‐ion‐based magneto‐ionic structures can be attained using a Li‐enriched LiPON layer without LCO. In this case, the magnetoionic multilayer operates as a supercapacitor. Because much lower amounts of Li ions migrate to and away from the magnetic film in this structure, the magnetic parameters are affected less but the ionic response time is greatly accelerated. For instance, we have demonstrated skyrmion nucleation, which requires small changes in PMA or DMI, in response to 60 μs voltage pulses.^[^ ^84^ ^]^ In this second case study, we assess the use of solid‐state magnetoionic supercapacitors for the emulation of synaptic behavior.

**Figure 8:**
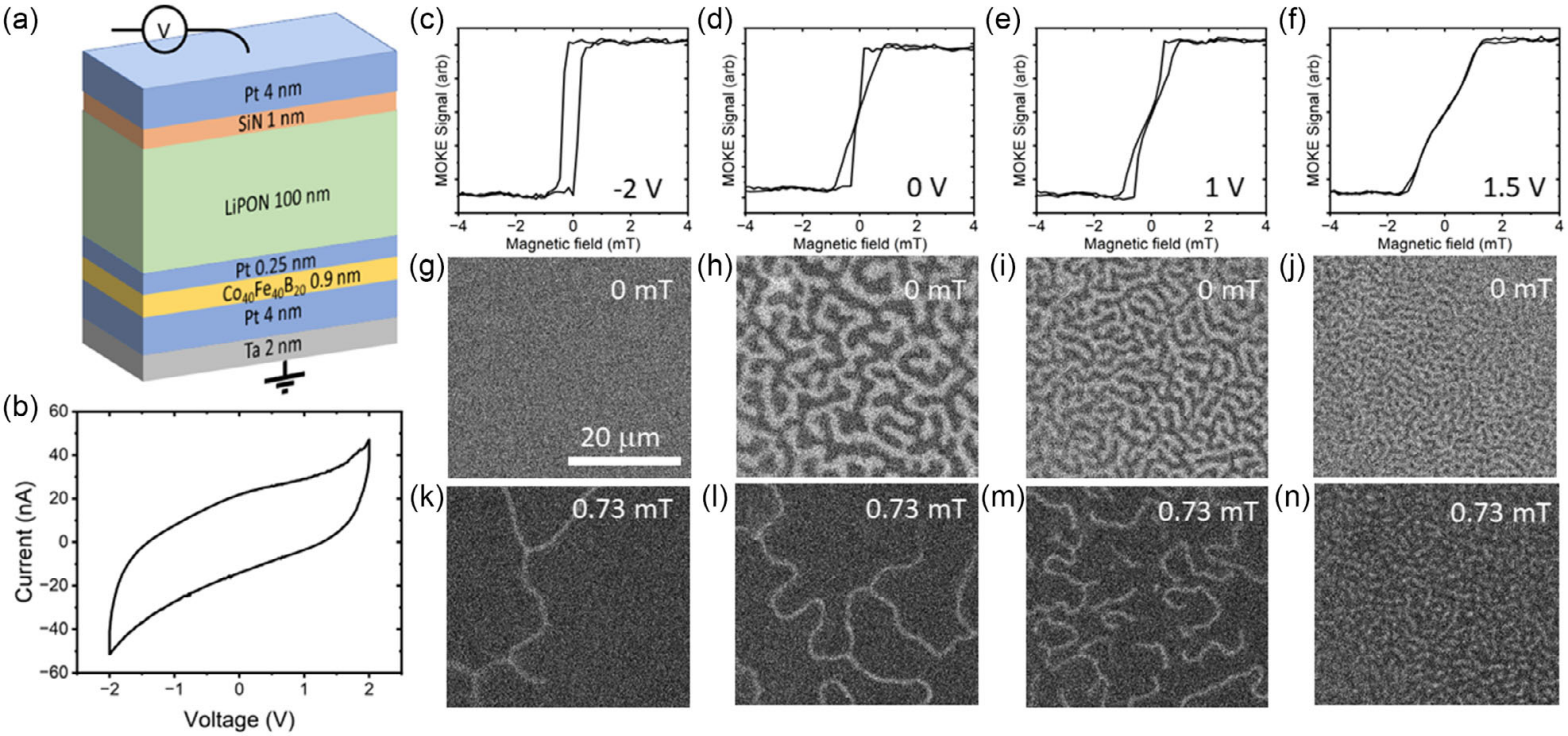
a) Cross‐sectional schematic of the sample. The bottom Ta/Pt electrode is grounded, and a voltage is applied to the top Pt contact. b) Cyclic voltammogram of a junction taken at a voltage sweep rate of 50 mV s^−1^. c–f) Polar MOKE hysteresis loops as a function of applied out‐of‐plane magnetic field for applied voltages of c) –2 V, d) 0 V, e) 1 V, and f) 1.5 V. g–j) Images with magnetic contrast obtained at 0 mT during the respective MOKE loop sweeps for the applied voltage indicated in the MOKE loop above each image. k–n) Images with magnetic contrast obtained at 0.73 mT during the MOKE loop sweeps at different applied voltages. All images are on the same scale, as indicated in (g).

Multilayer samples were grown using shadow‐masked magnetron sputtering to create 500 μm wide crossbar arrays of Ta (2)/Pt (4)/Co_40_Fe_40_B_20_ (0.9)/Pt (0.25)/LiPON (100 nm)/SiN (1)/Pt (4) (thicknesses in nm), where the top Pt layer was grown as a stripe at 90° to the underlying layers. A cross‐sectional schematic of the sample is shown in **Figure** **8**a. In this case study, we present data from different devices, with some device‐to‐device variation, but all showing the same essential features. The cyclic voltammogram shown in Figure 8b demonstrates that the electrical response of the sample is dominated by a capacitance with the contribution of a small leakage current. Peaks that would be indicative of redox reactions in the sample are absent. The device acts as a supercapacitor where mobile Li ions in the LiPON layer can form an EDL or intercalate at the LiPON/Pt/CoFeB interface under a driving voltage.^[^ ^84^ ^]^ The effect of this is seen in Figure 8c–f where polar magneto‐optic Kerr effect (MOKE) loops taken under different applied DC voltages show considerable variation depending on the applied voltage. At –2 V, where the Li ions are driven away from the CoFeB/Pt/LiPON interface, the MOKE loop is square, and the snapshot taken in zero magnetic field (Figure 8g) shows no domains. At 0 V (Figure 8d), the MOKE loop is no longer hysteretic around 0 mT, and the respective MOKE image (Figure 8h) shows a clear multidomain state. Increasing the voltage to 1 V, where Li ions are driven toward the CoFeB/Pt/LiPON interface, causes a further collapse in magnetic hysteresis (Figure 8e) and a denser multidomain state (Figure 8i), a trend which continues at 1.5 V (Figure 8f and j). In Figure 8k–n, the magnetic domain state at 0.73 mT after negative saturation is shown. As the voltage is increased from –2 to 1.5 V, two trends are seen. First, the domains become denser, consistent with a more slanted hysteresis loop, and second, the domains start to include skyrmions, with a few skyrmions visible at 1 V (Figure 8m) and a dense mixed skyrmion/stripe state occurring at 1.5 V (Figure 8n).

In the heterostructures studied here, the properties of the thin CoFeB layer are modified by the interfaces with Pt. The hybridization of the Co/Fe 3*d* orbitals with the Pt leads to two effects: first, PMA, which leads here to the magnetization preferentially lying out of the plane of the film, and second, the DMI, a form of chiral exchange which supports the existence of spiral magnetic textures such as helical domains and skyrmions.^[^ ^100^ ^]^ The effects of the applied voltage on the magnetic state are consistent with a reduction of the PMA or an increase in the DMI, which both act to stabilize a skyrmion state relative to uniform magnetization or stripe domain states. Previous experiments and density functional theory calculations have shown that the effect of intercalating Li ions in a Co/Pt bilayer is to reduce the PMA,^[^ ^75^ ^]^ without strongly changing the saturation magnetization. Moreover, the increasing density of skyrmions is consistent with the predictions of simulations for this system if the effect of voltage is to either reduce PMA or increase DMI.^[^ ^101^ ^]^

### 3.2 Emulation of Synaptic Behavior Using Magnetoionic Control of Stripe Domains and Skyrmions

**Figure 9:**
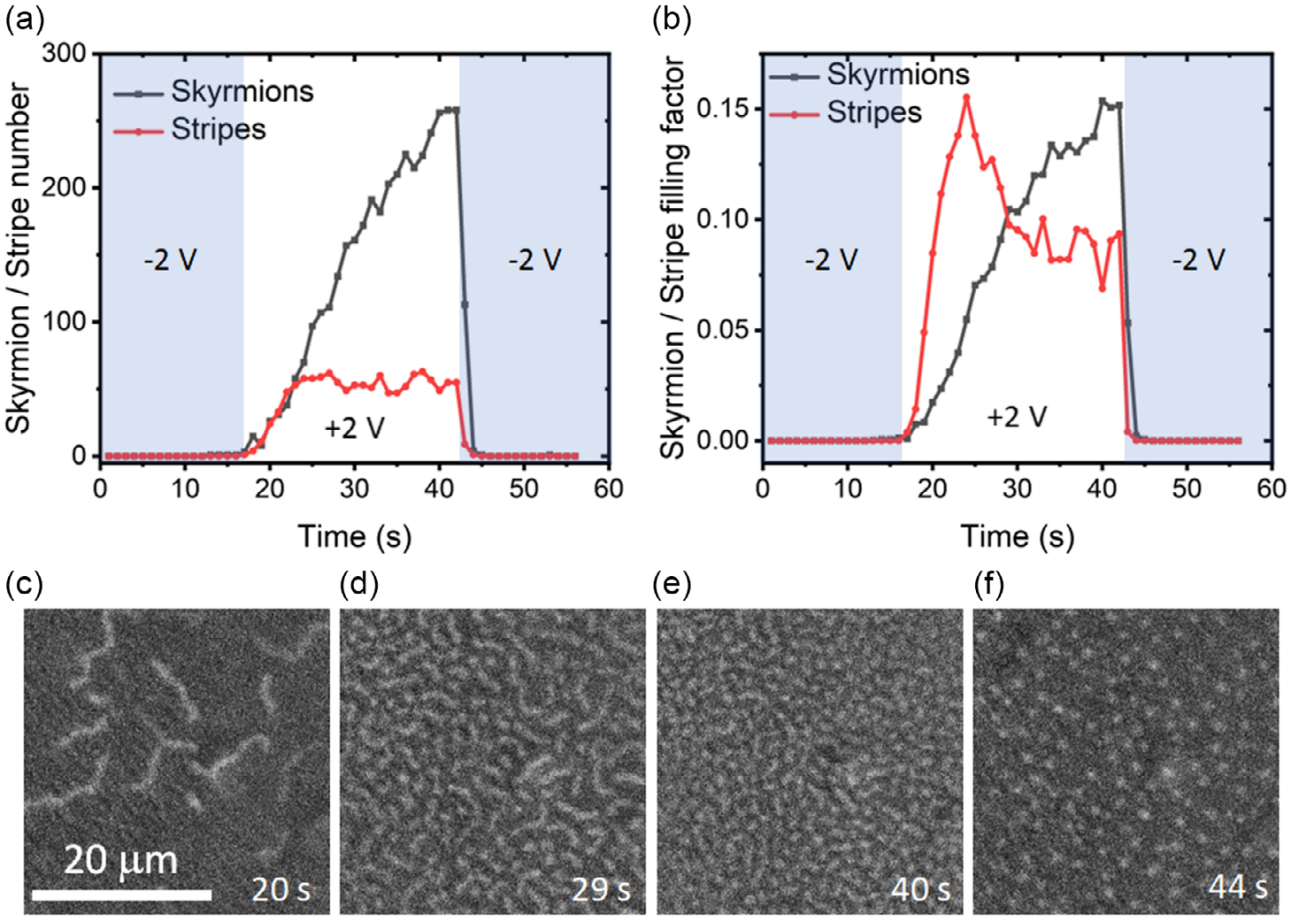
a) The number of skyrmions or stripe domains as a function of time through a set sequence of the applied voltage which switches from –2 to 2 V and back to –2 V. b) The fractional area filled by skyrmions and stripes for the same sequence of voltages as in (a). c–f) MOKE image snapshots of the domain states at the times indicated in the image which correspond to the data in (a,b). The scale is indicated in (c). The applied magnetic field in all measurements is 0.68 mT.

The effects on the magnetic state of stepping the voltage are shown in **Figure** **9**. Starting from an initial –2 V, a voltage step to 2 V is applied. In Figure 9a, the evolution of the number of stripe domains and skyrmions is shown, with the respective filling factor, that is, the fraction of the total area taken up by each domain, as shown in Figure 9b. The voltage step causes the magnetic domain state to evolve from an initial saturated state to a dense mixed stripe and skyrmion state. Shortly after the 2 V is applied, as shown in the magnetic snapshot in Figure 9c, there is nucleation of elongated stripe domains and a few skyrmions. Continuing the application of 2 V (Figure 9d) leads to a denser state with more skyrmions and shorter stripe domains. This turns into a state dominated by skyrmions (Figure 9e). Finally, the voltage is switched back to –2 V. This causes the remaining strip domains to annihilate first, causing the domain state to consist entirely of skyrmions (Figure 9f) before these are also annihilated. The changing net magnetization because of stripe domain and skyrmion nucleation/annihilation provides an example of potentiation and depression under continuously applied voltages. The existence of both stripe domains and skyrmions enables tailoring of the magnetic response time of the system as the stripe domains nucleate and annihilate on a faster timescale than the skyrmions. The different magnetic timescales provide a route to tuning the response of a neuromorphic device. Moreover, the thermally activated nucleation of stripe domains and skyrmions delivers the required stochasticity for emulating the behavior of biological synapses. To create a neuromorphic device from such a magnetoionic junction the magnetic state needs to be read out. This can be done through the anomalous Hall effect^[^ ^50^ ^]^ or by integrating the device with a magnetic tunnel junction.^[^ ^32^ ^]^ Either approach averages the magnetic signal over the junction area equating the synaptic weight to the fractional area with domains or skyrmions.

**Figure 10:**
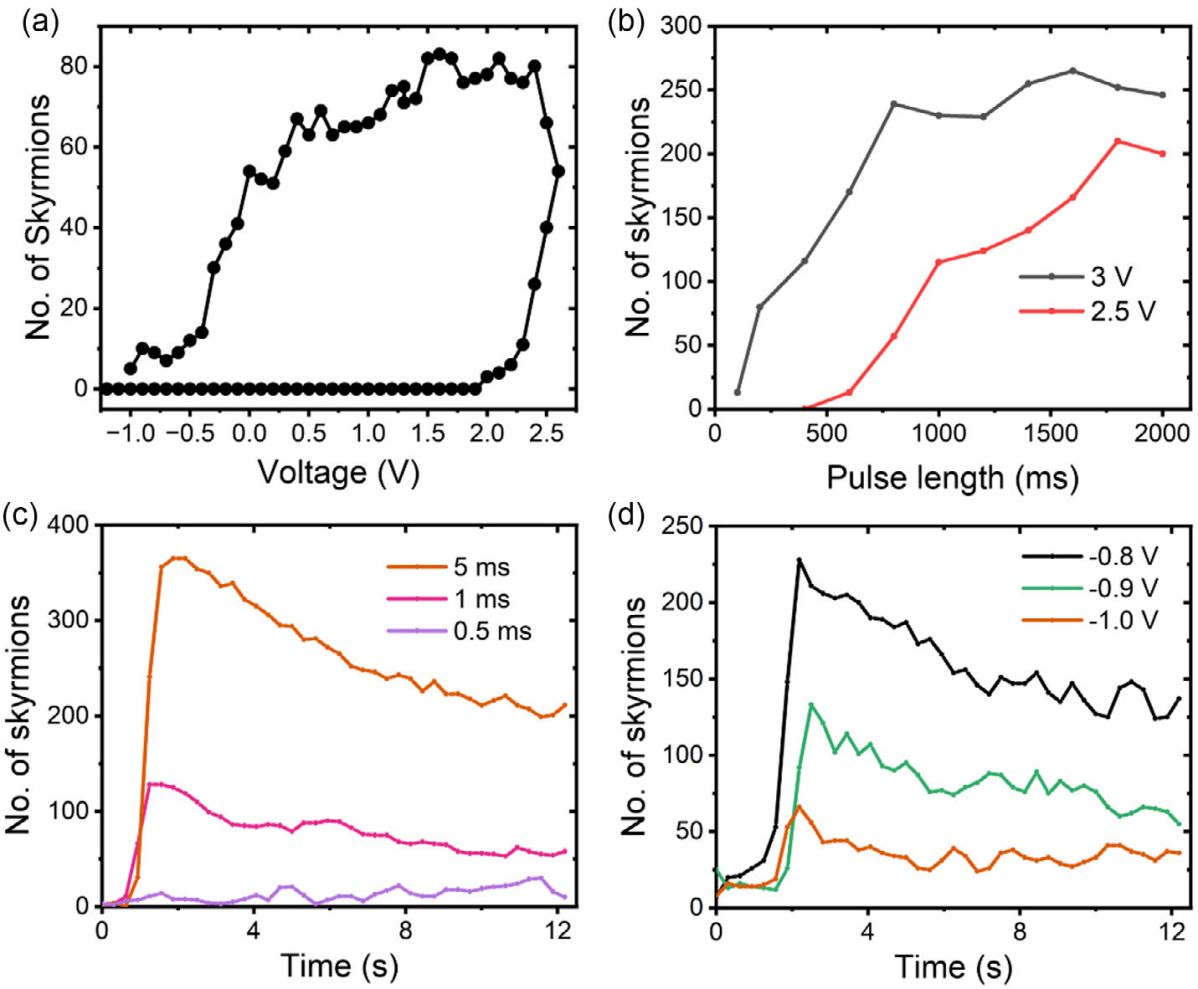
a) The number of skyrmions obtained using a stepped voltage under 0.7 mT applied magnetic field. The number of skyrmions is extracted from images taken 10 s after each voltage step. b) The number of skyrmions at 0 V after a voltage pulse from 0 to 2.5 V or 3 V as a function of the pulse length under 0.65 mT applied magnetic field. c) The number of skyrmions as a function of time following a voltage pulse applied from –1.1 V to +3 V and back to –1.1 V for different pulse length. The length of the voltage pulse is indicated in the legend and the applied magnetic field is 0.7 mT. d) The number of skyrmions as a function of time following a voltage pulse applied from different starting voltages, as shown in the legend, to +7 V for 400 μs, and back to the starting voltage. The applied magnetic field is 0.7 mT.

In **Figure** **10**a, we show the effect of a stepped voltage on the skyrmion number. The voltage is increased from negative voltages in steps of 0.1 V with the skyrmion number recorded 10 s after each step, reaching 2.6 V before being stepped back to negative voltages. The skyrmion number is strongly hysteretic, a feature demonstrating the required nonlinearity and memory for synaptic operation. In Figure 10b, the effects of applying ms pulses rather than a DC voltage to the sample are shown. Starting from a saturated state, a voltage is pulsed to either 2.5 or 3 V for a particular length of time before returning to 0 V. The number of skyrmions is recorded a second after the end of the pulse. In these pulsed experiments, we find that only skyrmion states are present one second after the pulse, which is consistent with the results of Figure 9 where the skyrmion states are more stable than stripe domains. This demonstrates three important features of the magnetoionic device. Firstly, the device can be placed into a voltage‐programmable final state obtained by tuning either the strength of the applied voltage or the length of the voltage pulse. Second, the ability to control the output state through the amplitude shows that protocols based on SADP can be applied to these devices. Third, there is a voltage threshold effect, which depends on the pulse length. For 400 ms pulse length, the 2.5 V pulse does not induce any skyrmions while applying a 3 V pulse of the same length induces a significant number of skyrmions.

In Figure 10c, the number of skyrmions recorded following pulses of different length is shown as a function of time. The junction is initialized in a skyrmion‐free state at –1.1 V then the voltage is pulsed to +3 V before returning to –1.1 V. Longer pulses lead to a greater number of nucleated skyrmions, with the shortest pulses having no strong effect on the sample, again showing a strongly nonlinear response to the input. The number of skyrmions peaks around 1 s after the application of the pulse, a consequence of the thermal activation of skyrmion nucleation. Following that, the number of skyrmions decays as the Li ions relax back to the equilibrium distribution in the LiPON layer at lower voltage. In Figure 10d we show the effect of changing the initial voltage from which the pulsed voltage is applied on the skyrmion nucleation and subsequent decay. The initial voltage is set as shown in the legend and then the voltage is pulsed to 7 V for 400 μs. Starting from a less negative initial voltage leads to a higher peak in the number of nucleated skyrmions after the pulse and a slower decay to higher equilibrium level afterward. This ability to tune both the magnitude of the magnetic response and the timescale of the decay would allow accurate control over the functioning of a synapse made from such a magneto‐ionic system.

The results of case study II show clear short‐term potentiation through positive voltage pulses as well as the plasticity of the response on the timescale of seconds. The timescale of the depression can be similarly controlled. Previously, it was shown that setting the device to 0 V after the nucleation of skyrmions leads to a decay time of around 8 min,^[^ ^84^ ^]^ while the data in Figure 9 show that setting the voltage to –2 V leads to a decay time of the order of a second. Taking these data into account along with the results shown here, it is clear that the response time of the magnetoionic device can be controlled by choosing both the average voltage level and the range of voltages applied to the junction. Both the potentiation and depression of the synaptic weight caused by a particular voltage pulse can be controlled. This ability to control the plasticity of the junction gives a wide‐ranging freedom in designing the input sequence of the synapse. These features come about through the interplay of the ionic timescales, determined by the transport of ions in LiPON, and the magnetic timescales determined by the nucleation and annihilation of magnetic domains and skyrmions.

## 4 Challenges, Opportunities, and Outlook

In summary, this perspective provides an overview of the potential use of magnetoionics for neuromorphic applications. Two examples of emerging magnetoionic synaptic systems have been described in detail. First, we have demonstrated the operation principle of a semiconducting Co_3_O_4_‐based magnetic synapse using oxygen ion migration. We have exploited the volatile and nonvolatile magnetoionic effects to mimic short‐term and long‐term synaptic plasticity, respectively. We have illustrated the physical mechanism governing these effects. Second, we have shown how magnetoionics can tailor the behavior of skyrmions, as an alternative way to modulate synaptic weight. Overall, while several effects have shown their own merit (as described in Sections 2 and 3), this research field is still at its early stages of development and there are several challenges to be addressed, both at the material level and for the implementation of devices.

Since magnetoionics relies on thermally activated ion diffusion processes, achieving fast switching rates at room temperature is one of the most important challenges. Several works have shown synaptic potentiation effects using pulses of ms.^[^ ^85^, ^87^ ^]^ In turn, skyrmion nucleation has been achieved in response to 60 μs voltage pulses in lithium‐based magnetoionic systems,^[^ ^84^ ^]^ and the generation of ferrimagnetic spin textures (chiral domain walls or skyrmions) through hydrogen gating in GdCo racetrack devices has been accomplished in times of 50 μs.^[^ ^102^ ^]^ Yet, reducing magnetoionic rates to sub‐μs would be desirable. This might be accomplished in ultrathin magnetoionic films and patterned dots, as well as mesoporous structures, where ion diffusion paths would become shorter and the strength of electric fields stronger. Relying on effects based on surface ion adsorption, rather than ion intercalation, would also be a promising approach to improve the speed. Along the same lines, solid‐state supercapacitor structures could be optimized further to quickly establish an EDL in the electrolyte without altering the structural and chemical integrity of the magnetic film.^[^ ^84^ ^]^ Besides improving the speed, the distinct magnetic and ionic timescales could be separately exploited in some systems, for instance, by simultaneous application of magnetic and electric fields. For example, the thermally activated nucleation of skyrmions occurs on a timescale which depends on the ionic response, which affects the magnetic parameters, but magnetic fields could be used to produce faster or slower responses. For applications in reservoir computing, the decay time of a state, which determines the memory of the system, is a key metric and should be notably longer than the frequencies of the input.

Another limiting factor in real applications is endurance. Some reports have shown magnetoionic switchability of the order of 10^3^–10^4^ cycles.^[^ ^73^, ^88^, ^102^ ^]^ While being close to the endurance of flash memories in solid‐state disks, these values are lower than in other electrically controlled devices, such as resistive random‐access memories (RE‐RAMs) or spin‐transfer‐torque magnetic random‐access memories (STT‐MRAMs).^[^ ^103^ ^]^ A prospective strategy to increase endurance could be to avoid phase transformations (e.g., redox reactions), resulting from ionic displacements. This has been demonstrated using the supercapacitor design in case study II where 750 000 electric cycles were achieved in a redox reaction‐free device.^[^ ^84^ ^]^ This could be further exploited by the mere insertion of small ions without heavily distorting the crystallographic structure of the target materials^[^ ^73^ ^]^ or through voltage‐driven formation/annihilation of vacancies in epitaxial films.^[^ ^88^ ^]^ An increase of cyclability has also been demonstrated through suitable electrolyte engineering.^[^ ^90^, ^104^ ^]^ Additionally, the use of light ion irradiation (keV He^+^, Ar^+^) to promote formation of vacancies or grain boundaries that act as effective ion diffusion paths (i.e., defect engineering) could boost both magnetoionic rates and endurance.^[^ ^105^, ^106^ ^]^

Challenges also exist in terms of device implementation. Crossbar arrays are typically used in neuromorphic layout architectures.^[^ ^107^ ^]^ Compared to memristors, the resistance of top gate oxides used in some magnetoionic systems is typically larger (GΩ) than in memristors (kΩ to MΩ). Thus, their power consumption can be lower for this standard 2D architecture. However, the biological brain is 3D. Thus, achieving selective magnetoionic actuation in 3D arrays of interconnected dots might be desirable to attain the necessary neuron/synapse densities to reproduce real cognitive functions. This could be prompted by the recent developments on the fabrication of functional 3D magnetic assembly/network architectures.^[^ ^108^, ^109^ ^]^ Moreover, magneto‐ionic synapses could exchange information between each other through magnetic interactions. Both exchange interactions, which can be direct Heisenberg exchange or indirect, for example RKKY interactions, can be modified by ionic insertion. An unpatterned magnetic mesa could be used to create an array of interacting neuron and synapse devices. Further long‐range dipolar fields could be used to create synaptic intercommunication between physically separated devices. Finally, magnetoionic effects using liquid electrolytes can be induced in a wireless manner,^[^ ^43^ ^]^ which may render new actuation procedures to mimic synaptic activity.

There are further ways in which magnetoionic devices may surpass two‐terminal memristors. First, due to the vectorial character of magnetization (as opposed to conductance, which is a scalar), it is possible to program magnetoionic synapses to exhibit both positive and negative synaptic weights (the latter yielding inhibitory postsynaptic potentials), without the need of additional circuitry. Notably, as in the case of skyrmion devices, an applied voltage creates a domain state where the magnetization directions cover the whole unit sphere. The ability of magnetoionic devices to convert from a low‐dimensional input to a high‐dimensional magnetic state is a feature that is readily exploitable for reservoir computing applications. Another attractive effect would involve the creation of a feedback mechanism between the magnetic and ionic parts of the skyrmion device. Presently, the ionic state affects the magnetic state but not vice versa. Incorporating magnetoresistive materials into magnetoionic junctions may offer feedback and increased device functionality for crossbar arrays.

Finally, the underlying working mechanisms of the human brain, as well as the physical and biochemical processes governing ion‐gated materials in general, are still far from being clear. Further research is needed on fundamental studies combining theory with in situ experiments, correlating structure and properties of materials. Merging the complementary backgrounds from neurobiologists, chemists, physicists, and materials engineers is required. Only in this way, further progress in the field of neuromorphic computation, and magneto‐ionic synaptic devices in particular, will be possible.

## Conflict of Interest

The authors declare no conflict of interest.
